# Comparison of the single-cell and single-nucleus hepatic myeloid landscape within decompensated cirrhosis patients

**DOI:** 10.3389/fimmu.2024.1346520

**Published:** 2024-02-06

**Authors:** Lukas Van Melkebeke, Jef Verbeek, Dora Bihary, Markus Boesch, Bram Boeckx, Rita Feio-Azevedo, Lena Smets, Marie Wallays, Eveline Claus, Lawrence Bonne, Geert Maleux, Olivier Govaere, Hannelie Korf, Diether Lambrechts, Schalk van der Merwe

**Affiliations:** ^1^ Laboratory of Hepatology, Department of Chronic Diseases and Metabolism, KU Leuven, Leuven, Belgium; ^2^ Department of Gastroenterology and Hepatology, University Hospitals Leuven, Leuven, Belgium; ^3^ Laboratory for Translational Genetics, Department of Human Genetics, KU Leuven, Leuven, Belgium; ^4^ VIB Center for Cancer Biology, Leuven, Belgium; ^5^ Department of Interventional Radiology, University Hospitals Leuven, Leuven, Belgium; ^6^ Department of Imaging and Pathology, Translational Cell and Tissue Research, KU Leuven and University Hospitals Leuven, Leuven, Belgium

**Keywords:** single cell sequence (scRNA-seq), single nucleus RNA sequencing, cirrhosis, transjugular biopsy, decompensated

## Abstract

**Background and aims:**

A complete understanding of disease pathophysiology in advanced liver disease is hampered by the challenges posed by clinical specimen collection. Notably, in these patients, a transjugular liver biopsy (TJB) is the only safe way to obtain liver tissue. However, it remains unclear whether successful sequencing of this extremely small and fragile tissue can be achieved for downstream characterization of the hepatic landscape.

**Methods:**

Here we leveraged in-house available single-cell RNA-sequencing (scRNA-seq) and single-nucleus (snRNA-seq) technologies and accompanying tissue processing protocols and performed an in-patient comparison on TJB’s from decompensated cirrhosis patients (n = 3).

**Results:**

We confirmed a high concordance between nuclear and whole cell transcriptomes and captured 31,410 single nuclei and 6,152 single cells, respectively. The two platforms revealed similar diversity since all 8 major cell types could be identified, albeit with different cellular proportions thereof. Most importantly, hepatocytes were most abundant in snRNA-seq, while lymphocyte frequencies were elevated in scRNA-seq. We next focused our attention on hepatic myeloid cells due to their key role in injury and repair during chronic liver disease. Comparison of their transcriptional signatures indicated that these were largely overlapping between the two platforms. However, the scRNA-seq platform failed to recover sufficient Kupffer cell numbers, and other monocytes/macrophages featured elevated expression of stress-related parameters.

**Conclusion:**

Our results indicate that single-nucleus transcriptome sequencing provides an effective means to overcome complications associated with clinical specimen collection and could sufficiently profile all major hepatic cell types including all myeloid cell subsets.

## Introduction

Cirrhosis represents a major cause of death worldwide and is characterized as the end stage of progressive liver fibrosis, in which the hepatic architecture is distorted, resulting in portal hypertension and loss of hepatic function (1). During the development of the advanced disease stage, cirrhosis is characterized by severe immune dysfunction and sustained systemic inflammation that may precipitate extrahepatic organ failure (2). Hepatic macrophages play a key role in this regard, as they contribute to both the progression and resolution of tissue inflammation (3). The recent application of single-cell RNA sequencing (scRNA-seq) and the development of a comprehensive human liver atlas have underscored the presence of a dense and diverse network of immune cells in the liver (3–5). In light of these findings, we can now recognize that the hepatic myeloid population is not exclusively composed of resident Kupffer cells but encompasses multiple populations of macrophages, even in a healthy state (4). Furthermore, during the development of cirrhosis, disease associated macrophage populations emerge that contribute to the maintenance of inflammation and the propagation of fibrosis (6–9). Moreover, spatial data enabled the identification and interaction of immune cells with other cells in their local environment, revealing signals that could direct niche-specific macrophage functions (9, 10).

Despite these advances, gaining a comprehensive understanding of human liver immune cells during advanced cirrhosis is significantly impeded by the challenges associated with clinical specimen collection and processing. For instance, fresh tissue necessitates immediate processing and enzymatic digestion, potentially resulting in the loss of sensitive or incompletely dissociated cells, along with alterations in gene expression. Moreover, the size of many structural hepatic cells may preclude their passage through microfluidic channels, leading to the non-recovery of their RNA cargo through this approach (11). Single-nucleus RNA-seq (snRNA-seq), on the other hand, could serve as an alternative strategy, involving the isolation of nuclei from frozen tissues, thereby circumventing the necessity for immediate sample processing. The drawback of this approach is that smaller subsets of cells, such as crucial macrophage subtypes, may be overshadowed by abundant structural cells. Nevertheless, snRNA-seq could still be the method of choice when dealing with specimens of extremely small size and fragility (12, 13).

In the context of investigating the hepatic landscape in decompensated cirrhosis, liver tissue can be safely obtained only through the transjugular route. However transjugular liver biopsies (TJBs) have an extremely small size ( ± 14.7mm^3^ compared to 80.4mm^3^ for 14G tru-cut needle biopsies), and successful sequencing of these specimens is yet to be demonstrated (14). In this study, we utilized in-house scRNA-seq and snRNA-seq technologies along with corresponding tissue processing protocols. We conducted a comparison of the data obtained from sequencing transjugular liver biopsies (TJBs) in patients with advanced cirrhosis. Importantly, we performed a within-patient comparison of both techniques to eliminate potential differences in cell subset frequencies arising from distinct disease states or stages among patients. Beyond assessing the global liver cell landscape, data quality, and cell recovery, we specifically examined how the transcriptomic profile of liver myeloid cells could be compared between the two techniques.

Our findings indicate that both scRNA-seq and snRNA-seq successfully identify myeloid cells from TJBs, with the gene signature of specific clusters and myeloid subpopulations being preserved between both techniques. However, the scRNA-seq platform failed to recover sufficient Kupffer cell numbers, and other monocytes/macrophages featured elevated expression of stress-related parameters. Moreover, our data offers valuable insights to consider when conducting sequencing experiments in the context of advanced cirrhosis.

## Patients and methods

### Patient population and sample collection

Liver biopsies were collected with ethics approval from the University Hospitals Leuven (ethical committee S64744) after written informed consent was given by the patient. From 3 patients with decompensated liver cirrhosis, a total of 5 TJBs per patient were taken with a standard 19G needle (Cook, Limerick, Ireland). The clinical characteristics are shown in **Supplementary Table 1**. All samples were immediately rinsed with an isotonic fluid. For each patient, half of the samples were snap frozen in liquid nitrogen (-196°C) for snRNA-seq at a later timepoint while the other half was placed in phosphate-buffered saline (PBS) for immediate transfer for cell isolation and scRNA-seq.

### Data availability statement

Raw sequence data has been deposited at the European Genome-phenome Archive (EGA), under accession number EGAS50000000073. This study did not generate any new code.

### Single-cell RNA-sequencing

Upon arrival, samples were rapidly processed for scRNA-seq. Samples were transferred to 2mL digestion medium containing collagenase P (2mg mL^−1^, ThermoFisher Scientific) and DNAse I (10U µL^−1^, Sigma) in DMEM (ThermoFisher Scientific). Samples were incubated for 15min at 37°C and pipetted up and down for 1min using a P1000 pipette. Next, 3mL ice-cold PBS was added, and samples were filtered using a 40µm nylon mesh (ThermoFisher Scientific). Following centrifugation at 300×*g* at 4°C for 5min, the supernatant was aspirated and discarded, and the cell pellet was resuspended in 1ml red blood cell lysis buffer (Roche). Following a 5min incubation at room temperature, samples were centrifuged (300×*g*, 4°C, 5min) and resuspended in 1mL PBS containing 0.04% Bovine Serum Albumin (BSA) and filtered over Flowmi 40µm cell strainers (VWR) using wide-bore 1mL low-retention filter tips (Mettler-Toledo). Next, 10µL of this cell suspension was counted using a LUNA-FL dual fluorescence cell (Logos Biosystems) counter to determine the concentration of live cells. Libraries for scRNA-seq were generated using the Chromium Single Cell 3’ library from 10x Genomics according to the manufacturers protocol. We aimed to profile 10,000 cells per library (if sufficient cells were retained during dissociation). The entire procedure, from the moment of biopsy until loading in the 10x Genomics device, was completed in <90 minutes. Afterwards, individual cells were emulsified and amplified with 3′ adaptors while attaching sample indices. Sequencing was performed using Novaseq 6000 (Illumina).

### Single-nucleus RNA-sequencing

Upon collection, samples were immediately snap frozen in liquid nitrogen. At processing the samples were placed in 1 mL of TST-buffer (**Supplementary Methods**) on a petri dish and chopped into small pieces using a scalpel followed by tissue homogenization with a Dounce homogenizer (i.e., during 2.5 min with the loose pestle and 2.5 min with the tight pestle). The homogenized solution was then filtered through a 40 µm cell strainer (Falcon) and placed in a 50 mL Falcon tube. Afterwards the filter was washed once with 0.5 mL of ST-buffer (**Supplementary Methods**). This process was repeated on a 20 µm, a 10 µm and a 5 µm cell strainer (Falcon). Afterwards, the sample was transferred to a 15mL Falcon tube before being centrifuged at 4°C for 5min at 500g. The pellet was resuspended in 300µL of PBS containing 1.0% of BSA. Next, 10µL of this cell suspension was counted using a LUNA-FL dual fluorescence cell counter (Logos Biosystems) to determine the concentration of the nuclei. Libraries for snRNA-seq were generated using the Chromium Single Cell 3’ library from 10x Genomics according to the manufacturers protocol. We aimed to profile 20,000 nuclei per library. The entire procedure was completed in <60 min. Afterwards, individual nuclei were emulsified and amplified with 3′ adaptors while attaching sample indices. Sequencing was performed using Novaseq 6000 (Illumina).

### Data analysis

#### General statistics

Normally distributed data are reported as mean ± standard deviation, while non-normally distributed data are reported as median with interquartile range. Normality was tested using the Shapiro-Wilk test. The proportion and absolute numbers of cells and nuclei, the number of genes per cell/nuclei and the number of counts per cell/nuclei were compared using a paired sample t-test or Wilcoxon matched-pairs signed ranked test according to the type of data. Significance was defined as a two-sided p<0.05. Statistical analysis and graphs were produced using GraphPad Prism v9.0 (GraphPad Software) or the respective R v4.1.2 packages.

#### Preprocessing of scRNA-seq and snRNA-seq data

Raw sequencing reads were aligned to the human reference genome (GRCh38/hg38) and gene-expression matrices were generated with CellRanger (v3.0.2). Gene-cell matrix was used as input in Seurat (v4.1.1) for analysis (15). Genes expressed in less than three cells were excluded. The estimated ambient contamination fraction was calculated using SoupX (v1.6.2). The count matrix was filtered for cells exhibiting 800-8,000 genes as well as <30% mitochondrial genes of the total UMI counts (a comparable percentage as in other papers in the field of hepatology (8, 11)). Doublets were identified using DoubletFinder (v2.0.3) (16). Samples were log-normalized with a scale factor of 10,000 and anchor integrated. The variation between cells in UMI counts and mitochondrial gene content was regressed out. The number of reads and saturation per sample is stated in **Supplementary Table 2**.

#### Dimensionality, clustering and differential gene expression

Unsupervised clustering and differential gene expression analysis were performed in Seurat. Clustering on the transjugular liver biopsy samples was done using shared nearest neighbors with 30 principal components based on integrated dataset variability shown in principal component analysis (PCA). Louvain clustering with a resolution of 2 was used to determine the number of clusters. Next, clusters were combined and labeled based on published marker genes (8, 11, 17). Doublet clusters were identified using the DoubletScore and removed. Low quality clusters were identified based on differentially expressed genes and removed. Several cell types were sub-clustered for further analysis. Each new Louvain clustering and uniform manifold approximation and projection (UMAP) reduction utilized dimensions between 10 and 20 for regression. Markers for each sub-cluster were identified with the FindAllMarkers functions, and cell types were manually annotated. Seurat was used together with ggplot2 (v3.3.6) and pheatmap (v1.0.12) packages to generate heatmaps, violin plots, barplots, dotplots and UMAP visualizations. Differential gene analysis in Seurat was performed with Wilcoxon Rank Sum with genes only present in at least 25% of cells. A correlation prediction score was calculated in the snRNA-seq and scRNA-seq dataset using the FindTransferAnchors and TransferData functions of the Seurat R Package using the scRNA-seq or sn-RNAseq as reference dataset respectively.

#### Comparison of scRNA-seq and snRNA-seq

We performed differential abundance analysis on the clusters and subclusters derived from different techniques, utilizing the miloR package (v1.2.0) to build a kNN graph (k=30, d=15-30) and define cell neighbourhoods (prop=0.2) (18). Neighbourhoods were quantified using countCells after calculating their distance with calcNhoodDistance (d=15-30). The differential abundance was evaluated with testNhoods across the conditions and visualized in a barplot. Significance was defined as an adjusted, two-sided p-value of <0.05. Spearman correlation was computed for the log2-transformed gene expression profiles of the cell and nucleus data (RNA-assay), specifically for the protein-coding genes. The set of protein coding human genes was downloaded from the Ensembl database (GRCh38.p14) using the BiomaRt R package. Gene set scores were calculated using the Addmodulescore function in Seurat and compared using a Wilcoxon-rank sum test. Single sample gene set enrichment analysis (ssGSEA) was performed using the R package GSVA (v1.46.0), and exporting the HallMark, KEGG, Reactome and Gene ontology (GO) gene sets from the MsigDB (v7.4) database using the R package GSEABase (v1.60.0). Limma (v3.54.2) was utilized to identify significantly (adjusted p-value <0.05, t-score >4) enriched gene sets across the calculated gene set scores. Plots were generated with ggplot2.

## Results

### SnRNA-seq and scRNA-seq of TJBs differentially recover all major hepatic cell types

**Figure 1:**
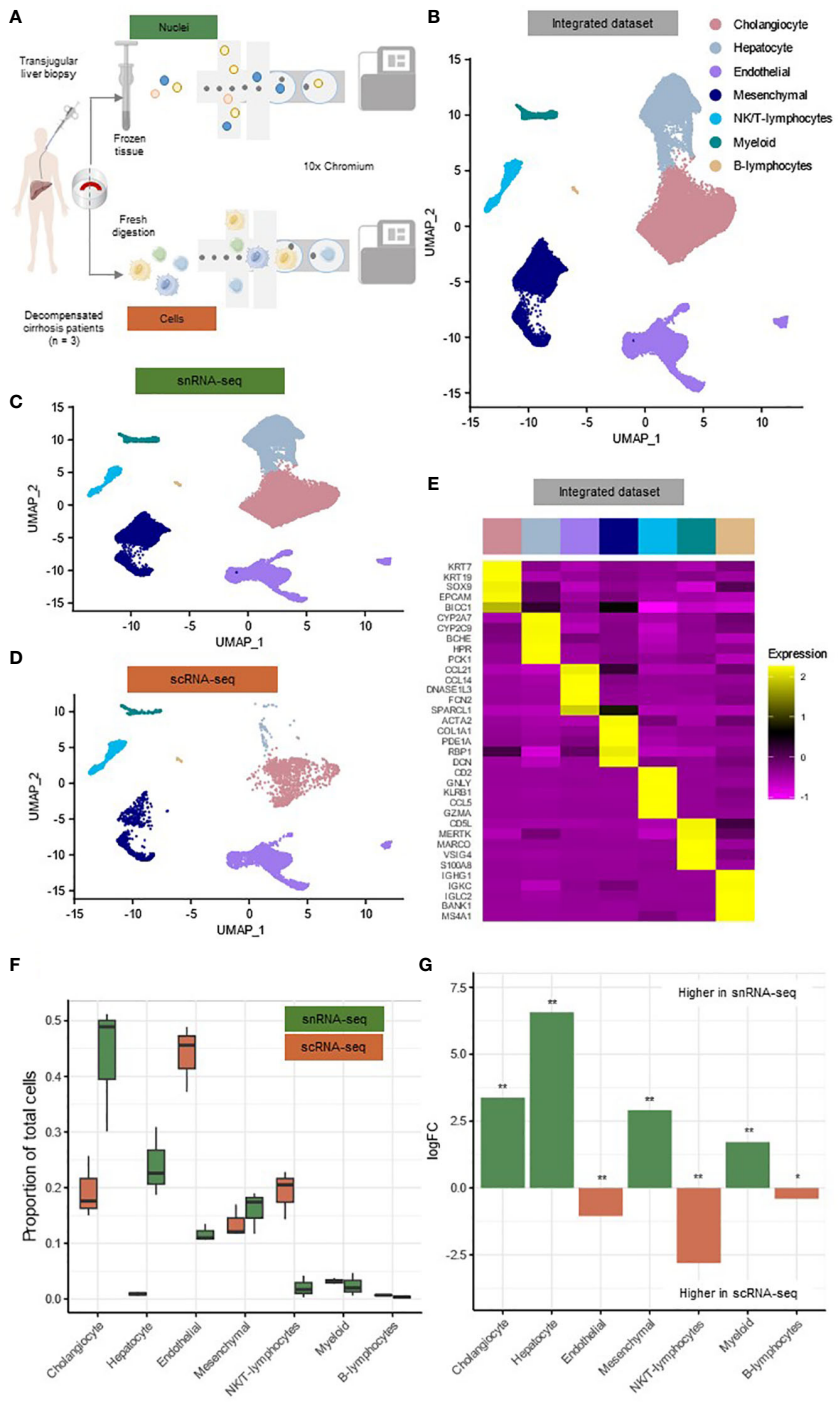
SnRNA-seq and scRNA-seq of TJBs differentially recover major hepatic cell types. **(A)** Depiction of the experimental design workflow. **(B)** Annotated UMAP plot of 31,410 single nuclei and 6,152 single cells, showing the different cell types. **(C)** Annotated UMAP plot of 31,410 single nuclei, showing the different cell types. **(D)** Annotated UMAP plot of 6,152 single cells, showing the different cell types. **(E)** Heatmap showing marker gene expression for the cell types of the full dataset. **(F)** Boxplot showing the percentage of every cluster in each sample. **(G)** Barplot showing mean logFC per cell type as calculated using MiloR. P-value adjusted for multiple testing being the minimum SpatialFDR. * p_adj_<0.05, ** p_adj_<0.01. *scRNA-seq, single-cell RNA-sequencing; snRNA-seq, single-nucleus RNA-sequencing; UMAP, uniform manifold approximation and projection*.

SnRNA-seq and scRNA-seq was performed on transjugular liver biopsy material from the same patient (n = 3) (patients characteristics included in **Supplementary Table 1**). Hereby half of the material was snap-frozen and subjected to snRNA-seq, while the other half was immediately processed for scRNA-seq (**Figure 1A**). Both single-cell and single-nucleus libraries were prepared using the 10X Genomics platform. Following data integration, quality filtering and clustering analysis, we identified 31,410 single nuclei (10,470 ± 3,615 per sample) and 6,152 single cells (2,051 ± 1,204 per sample) for the two techniques, respectively (**Figure 1B**). Cells and nuclei were well integrated across the different patients (**Supplementary Figures S1A, B**).

Based on previously canonical described marker genes, all major hepatic cell types were identified in the integrated dataset of single cells and nuclei and in both datasets separately (**Figures 1B–D**; **Supplementary Figures S1C, D**) (8, 11, 17). (0.4%) These main clusters along with their signature genes include cholangiocytes (*KRT7, KRT19, SOX9*) (41.4%), hepatocytes (*CYP2A7, CYP2C9, BCHE*) (19.1%), endothelial cells (*CCL21, CCL14, FCN2)* (17.0%), mesenchymal cells (*ACTA2, COL1A1, PDE1A*) (15.6%), NK/T-lymphocytes (*CD2, GNLY, KLRB1*) (4.6%), myeloid cells (*MERTK, MARCO, VSIG4*) (2.3%) and B-lymphocytes (*IGHG1, IGKC, IGLC2*) (0.4%) (**Figures 1B, E**, **Supplementary List 1**). All major cell types were present in both techniques for every individual patient and formed separate clusters when integrated for each individual technique (**Figures 1C, D**; **Supplementary Figure S1C, D**). However only a limited amount of hepatocytes was present in scRNA-seq (n=58; 0.9%) compared to snRNA-seq (n=7,132; 22.7%).

To interrogate the frequencies of the cell types retrieved by both techniques, we implemented the MiloR package (**Figures 1F, G**) (18). This package was specifically designed for differential abundance testing in single-cell datasets and was tested on data from human liver biopsies to outperform alternative methods (18). In the snRNA-seq dataset, we detected a more abundant number of cholangiocytes (mean logFC=3.37, p_adj_<0.01), hepatocytes (mean logFC=6.57, p_adj_<0.01), mesenchymal cells (mean logFC=2.91, p_adj_<0.01) and myeloid cells (mean logFC=1.71, p_adj_<0.01) (**Figure 1G**). On the other hand, elevated frequencies of endothelial cells (mean logFC=1.04, p_adj_<0.01), NK/T-lymphocytes (mean logFC=2.81, p_adj_<0.01) and B-lymphocytes (mean logFC=0.41, p_adj_<0.05) were present in the scRNA-seq dataset (**Figure 1G**).

### Comparison of the hepatic myeloid landscape detected by snRNA-seq or scRNA-seq

**Figure 2:**
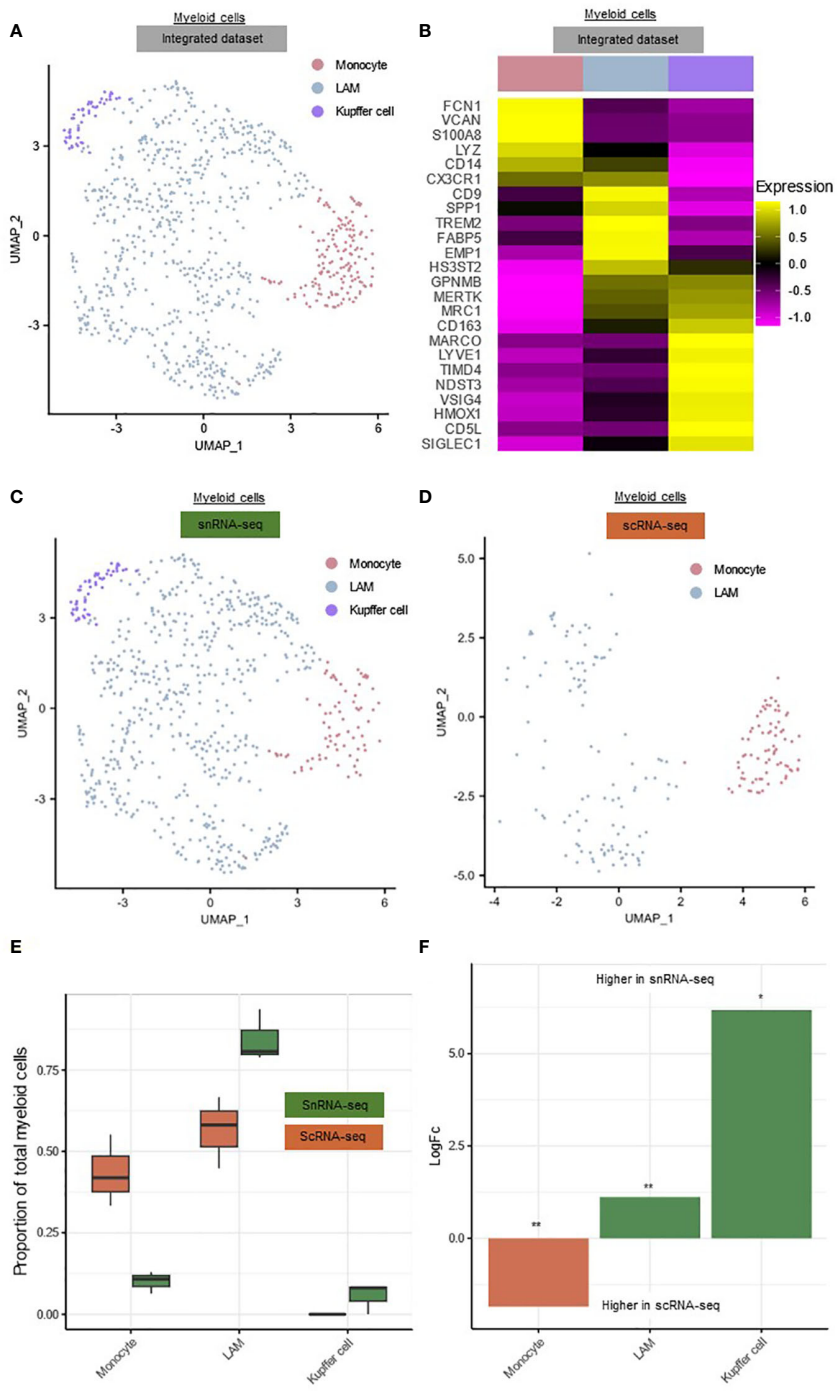
Comparison of the hepatic myeloid landscape detected by snRNA-seq or scRNA-seq. **(A)** Annotated UMAP plot of 689 single nuclei and 187 single cells of the myeloid cells, showing the different subpopulations. **(B)** Heatmap showing marker gene expression for the subclusters of the myeloid cells. **(C)** Annotated UMAP plot of 689 single nuclei of the myeloid cells, showing the different subpopulations **(D)** Annotated UMAP plot of 187 single cells of the myeloid cells, showing the different subpopulations **(E)** Boxplot showing the percentage of every myeloid subpopulation in each sample. **(F)** Barplot showing mean LogFC per subpopulation of the myeloid cells, as calculated using MiloR. P-value adjusted for multiple testing being the minimum SpatialFDR. * p_adj_<0.05, ** p_adj_<0.01, *scRNA-seq, single-cell RNA-sequencing; snRNA-seq, single-nucleus RNA-sequencing; UMAP, uniform manifold approximation and projection*.

We next performed a deeper analysis of the myeloid cell clusters and their frequencies within the integrated scRNA-seq and snRNA-seq datasets. We observed the presence of three transcriptionally distinct liver myeloid cell subpopulations (**Figures 2A, B**, **Supplementary List 1**). The first subset was characterized by the expression of markers associated with liver resident macrophages (Kupffer cells (5.9%)) (*MARCO, NDST3, TIMD4*) (**Figures 2A, B**) (3). The second cluster we could identify, expressed genes such as *CD9, SPP1, TREM2*, reminiscent of lipid-associated macrophages (LAM, 76.5%) (**Figures 2A, B**) (3). We further detected a population of monocytes typically expressing markers such as *FCN1, S100A8, VCAN* (17.6%) (**Figures 2A, B**) (3). The low percentage of Kupffer cells and high percentage of LAMs in patients with advanced liver disease is in line with the observations of previous reports (3, 9).

The cellular retrieval of the myeloid subpopulations in both techniques differed notably (**Figures 2C-F**). In snRNA-seq, only 10.06 ± 3.40% of all nuclei were monocytes, compared to 43.45 ± 11.01% of all scRNA-seq cells (p=0.052) (**Figure 2E**). On the other hand, scRNA-seq failed to recover any Kupffer cells (**Figure 2E**). MiloR analysis showed that LAMs (mean logFC=1.12, p_adj_<0.01) and Kupffer cells (mean logFC=6.18, p_adj_<0.05) were significantly more abundant in snRNA-seq, while monocytes (mean logFC=1.85, p_adj_<0.01) were significantly more present in scRNA-seq (**Figure 2F**). Because scRNA-seq was not able to detect Kupffer cells and retrieved less macrophages, this technique seems less suitable for studying the hepatic myeloid cell landscape in TJB from decompensated cirrhosis patients.

### Comparison of non-myeloid subclusters detected by snRNA-seq or scRNA-seq

**Figure 3:**
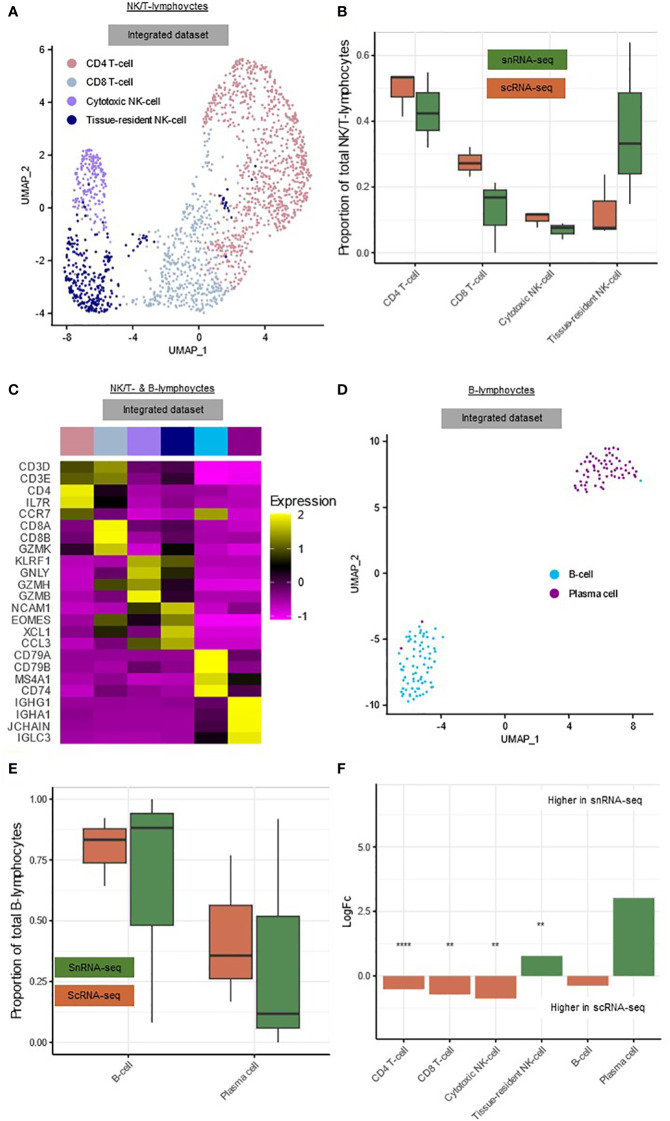
snRNA-seq and scRNA-seq differentially detect subclusters of hepatic lymphocytes. **(A)** Annotated UMAP plot of 573 single nuclei and 1,143 single cells of the NK/T-lymphocytes, showing the different subclusters. **(B)** Boxplot showing the percentage of every NK/T-lymphocyte subpopulation in each sample. **(C)** Heatmap showing marker gene expression for the subclusters of the NK/T- and B-lymphocytes. **(D)** Annotated UMAP plot of 99 single nuclei and 46 single cells of the B-lymphocytes, showing the different subclusters and techniques. **(E)** Boxplot showing the percentage of every B-lymphocyte subpopulation in each sample. **(F)** Barplot showing mean LogFC per subcluster of the immune cells, as calculated using MiloR. P-value adjusted for multiple testing being the minimum SpatialFDR. ** p_adj_<0.01, **** p_adj_<0.0001. *scRNA-seq, single-cell RNA-sequencing; snRNA-seq, single-nucleus RNA-sequencing; UMAP, uniform manifold approximation and projection*.

The NK/T-lymphocytes could be subclustered into CD4^+^ T-cells (*CD3D, CD3E, CD4*) (49.5%), CD8^+^ T-cells (*CD3D, CD3E, CD8A, CD8B*) (24.1%), cytotoxic NK-cells (*GNLY, GZMB, KLRF1*) (10.0%) and tissue-resident NK-cells (*EOMES, NCAM1, XCL1*) (16.4%) (**Figures 3A–C**, **Supplementary List 1**) (19). Due to the limited total number of NK/T-lymphocytes, these subclusters were not subdivided further. The B-lymphocyte cluster could be subclustered into B-cells (*CD79A, CD79B, MS4A1*) (52.4%) and plasma cells (*IGHG1, IGHA1, JCHAIN*) (47.6%) (**Figures 3C–E**, **Supplementary List 1**).

Within the NK/T-lymphocytes, tissue-resident NK-cells (mean logFC=0.78, p_adj_<0.01) were significantly more abundant in snRNA-seq, while CD4^+^ T-cells (mean logFC=0.52, p_adj_<0.0001), CD8^+^ T-cells (mean logFC=0.72, p_adj_<0.01) and cytotoxic NK-cells (mean logFC=0.87, p_adj_<0.01) were significantly more abundant in scRNA-seq (**Figure 3F**). In the B-lymphocytes there were no significant differences in abundance, this could however be caused by the low number of cells and nuclei (**Figure 3F**).

**Figure 4:**
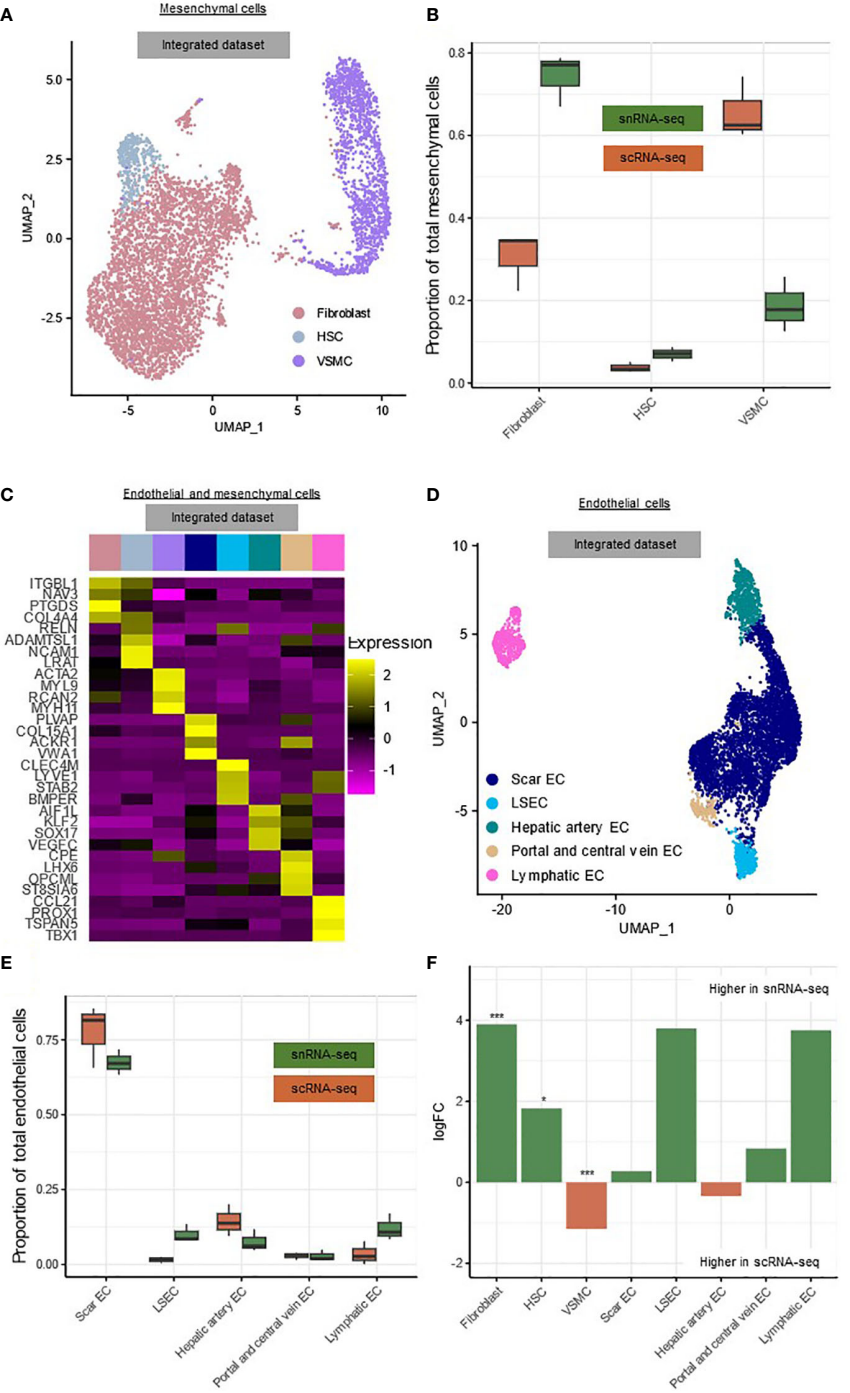
snRNA-seq and scRNA-seq differentially detect subclusters of mesenchymal cells. **(A)** Annotated UMAP plot of 5,023 single nuclei and 814 single cells of the mesenchymal cells, showing the different subclusters. **(B)** Boxplot showing the percentage of every mesenchymal subpopulation in each sample. **(C)** Heatmap showing marker gene expression for the subclusters of the mesenchymal- and endothelial cells. **(D)** Annotated UMAP plot of 3,666 single nuclei and 2,707 single cells of the endothelial cells, showing the different subclusters. **(E)** Boxplot showing the percentage of every endothelial subpopulation in each sample. **(F)** Barplot showing mean logFC per subcluster of the mesenchymal and endothelial cells, as calculated using MiloR. P-value adjusted for multiple testing being the minimum SpatialFDR. * p_adj_<0.05, *** p_adj_<0.001. *scRNA-seq, single-cell RNA-sequencing; snRNA-seq, single-nucleus RNA-sequencing; UMAP, uniform manifold approximation and projection*.

The mesenchymal cells could be subdivided into fibroblasts (FB) (*COL4A4, NAV3, PTGDS*) (68.7%), hepatic stellate cells (HSC) (*ADAMTSL1, LRAT, RELN*) (6.2%) and vascular smooth muscle cells (VSMC) (*MYL9, ACTA2, MYH11*) (25.0%) (**Figures 4A–C**, **Supplementary List 1**) (8, 9, 11). As expected, HSCs and FBs clustered together because they have similar phenotypes, while the VSMCs clustered separately (**Figure 4A**) (8, 9, 11). We observed a high number of FBs compared to HSCs, which is in line with other data reported from cirrhotic human livers (8, 11). The endothelial cells could be subdivided into scar-associated endothelial cells (scarEC; *COL15A1, PLVAP, VWA1*) (72.2%), liver sinusoidal endothelial cells (LSEC; *CLEC4M, LYVE1, STAB2*) (5.9%), hepatic artery endothelial cells (*AIF1L, KLF2, SOX17*) (10.4%), venous endothelial cells (*CPE, LHX6, OPCML*) (2.8%) and lymphatic endothelial cells (*CCL21, PROX1, TSPAN5*) (8.7%) (**Figures 4C–E**, **Supplementary List 1**) (8, 9, 17). The number of scarECs was elevated compared to the number of LSECs, as was reported previously in human cirrhotic livers (**Figure 4E**) (8, 11).

Within the mesenchymal cells, scarECs (mean logFC=3.90, p_adj_<0.001) and LSECs (mean logFC=1.83, p_adj_<0.05) were significantly more abundant in snRNA-seq, while VSMCs (mean logFC=1.15, p_adj_<0.001) were significantly more abundant in scRNA-seq (**Figure 4F**). There were no significant differences between both techniques for the endothelial cell subclusters (**Figure 4F**).

### Comparison of gene signatures in snRNA-seq and scRNA-seq

**Figure 5:**
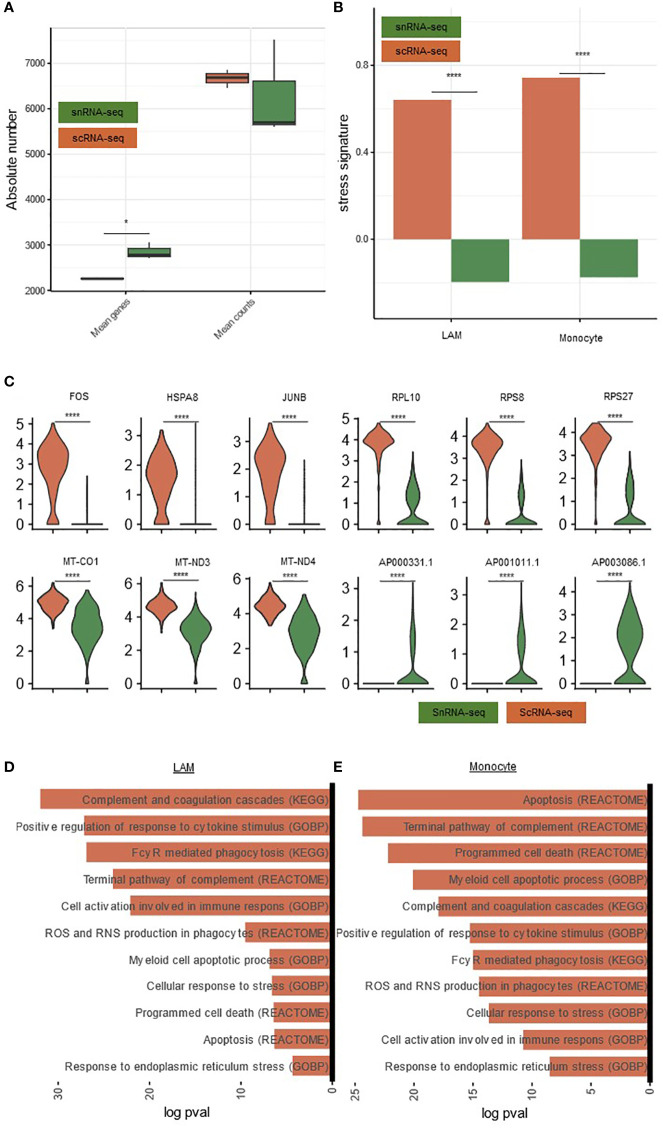
Comparison of gene expression and gene signatures in snRNA-seq and scRNA-seq. **(A)** Boxplots comparing scRNA-seq and snRNA-seq for mean genes and counts per sample. P-value calculated with a paired t-test. **(B)** Barplot comparing the stress signature between both techniques in monocytes and LAMs. Significance calculated using a Wilcoxon rank-sum test **(C)** Violin plot showing the expression of stress-related, ribosomal, mitochondrial and long non-coding RNA, comparing the snRNA-seq data with the scRNA-seq data. **(D)** Pathways significantly upregulated in scRNAseq compared to snRNA-seq in LAMs. **(E)** Pathways significantly upregulated in scRNA-seq compared to snRNA-seq in Monocytes. * p_adj_<0.05, **** p_adj_<0.0001 *scRNA-seq, single-cell RNA-sequencing; snRNA-seq, single-nucleus RNA-sequencing*.

After comparing both techniques for cluster and subcluster recovery, we next evaluated both techniques in terms of cell/nuclei recovery and gene signatures. The mean number of genes detected per nuclei/cell (2,851 ± 188 vs. 2,260 ± 16, p<0.05) was significantly elevated in the snRNA-seq dataset (**Figure 5A**). The number of counts per nuclei/cell was comparable between both techniques (6,274 ± 1082 vs. 6,669 ± 201, p NS) (**Figure 5A**).

To investigate if the core marker genes from the different subsets were comparable between both techniques, we used the gene signature of the single cells to predict the clustering of the single nuclei, and vice versa, using Seurat. Based on the gene signature of scRNA-seq and snRNA-seq, we could correctly identify the major cell types of 98.8% of the nuclei and 99.8% of the cells respectively (**Supplementary Figures S2A, B**). Specifically for the myeloid cluster, 99.4% of the nuclei and 99.5% of the cells were correctly predicted. This with a high mean correlation prediction score, as a measure for the certainty of the prediction, of 99.2 and 99.4 respectively, showing that the gene signature of myeloid cells was preserved in both techniques (**Figures S2A, B**).

In the myeloid subpopulations, we focused on the monocytes and LAMs since there were no Kupffer cells retrieved in scRNA-seq. For the monocytes, 100% of all nuclei and 77.6% of all cells were predicted correctly, with reasonable to good mean correlation prediction scores of 95.2 and 71.3 respectively (**Supplementary Figures S2A, B**). For the LAMs, 91.9% of all nuclei and 98.2% of all cells were predicted correctly, with good mean correlation prediction scores of 87.0 and 90.3 respectively (**Supplementary Figures S2A, B**). In addition, cell type intrinsic (pseudobulk) profiles of protein-coding genes were overall similar between snRNA-seq and scRNA-seq (Spearman correlation ρ = 0.75 in LAM and 0.73 in monocytes) (20). We calculated this in the myeloid subclusters (and not myeloid cluster), to minimize the effect of the differential abundance of specific subclusters in both techniques on the gene expression. This shows that also for the specific myeloid subpopulations, the gene signature was largely preserved in both techniques.

Nevertheless, also some important differences could be detected. In this regard, the transcriptomic data set from cells featured an elevated dissociation-induced stress signature (e.g. *FOS, HSPA8, JUNB*) (**Figures 5B, C**) as well as the expression of ribosomal (e.g. *RPL10, RPS8, RPS27*) and mitochondrial (e.g. *MT-CO1, MT-ND3, MT-ND4*) genes, similar to previous reports (20). In turn, nuclei exhibited elevated levels of long non-coding RNA (e.g. *AP000331.1, AP001011.1, AP003086.1*) (**Figure 5C**) (20–22). Furthermore, in a pathway analysis, immune-cell activation, apoptosis-related, phagocytosis, complement and stress-related pathways were increased in scRNA-seq compared to snRNA-seq, both in LAMs and monocytes (**Figures 5D, E**). Importantly, we also observed an elevated dissociation-induced stress signature in all other major clusters (**Supplementary Figure S2C**). This is compatible with an increased dissociation-induced stress in scRNA-seq.

## Discussion

The capacity to examine advanced liver disease at the single-cell level could significantly enhance our comprehension of the pathophysiology in disorders such as acute-on-chronic liver failure (ACLF), severe alcoholic hepatitis, and decompensated cirrhosis. It is crucial to acknowledge that in these advanced disease states, obtaining liver tissue safely is only possible through the transjugular route, resulting in extremely small and fragile liver biopsy specimens (14, 23). Currently, it remains uncertain whether successful sequencing of TJBs can be achieved for downstream characterization of the hepatic landscape. Therefore, we conducted an in-patient comparison of snRNA‐seq and scRNA‐seq protocols on TJBs obtained from 3 decompensated cirrhosis patients.

By following the appropriate protocol, we were able to consistently obtain approximately 10,000 single nuclei and 2,000 high-quality single cells per patient. Although the number of nuclei fell within the expected range, the count of cells tended to be lower than reported in the literature, likely attributable to the small size of the transjugular liver biopsy (11, 24). The data unveiled several differences that align with findings from comparable studies on the healthy human liver using whole liver lobes (11, 17). In particular, the snRNA-seq data showed a higher percentage of parenchymal and mesenchymal cells, but a lower percentage of endothelial cells and lymphocytes, in comparison to scRNA-seq (11). Similar to other studies on human liver biopsies, scRNA-seq revealed impaired recovery of hepatocytes, in contrast to snRNA-seq (8, 9). The reduced number of hepatocytes may be attributed to their vulnerability, rendering them susceptible to cell death during enzymatic dissociation. Alternatively their size could hinder their passage through the microfluidic channels. Furthermore, we observed significant differences in the retrieval of subclusters, such as a higher frequency of hepatic stellate cells (HSCs) and fibroblasts in snRNA-seq compared to a higher frequency of vascular smooth muscle cells (VSMCs) in scRNA-seq. We propose that dissimilarities in cellular retrieval between the two techniques stem from variations in the dissociation protocols employed. In the case of snRNA-seq, where the goal is to exclusively recover nuclei, a robust mechanical dissociation protocol can be employed. Conversely, for scRNA-seq, the requirement for live cells during library preparation necessitates the use of a gentler enzymatic dissociation protocol. This method tends to selectively favour resilient and/or more easily dissociable cell types, such as lymphocytes.

Overall, we found that snRNA-seq performed well in terms of sensitivity and classification of all hepatic cell (sub)-types and exhibited less cell type bias, as has been observed in other types of human tissues (24, 25). On the other hand, scRNA-seq emerges as the preferred platform for investigating lymphocytes, benefitting from its distinct positive selection of these cells. This preference is further underscored by the low level of B- and T-cell receptor transcripts in sn-RNAseq data, a finding that is consistent with that of Andrews et al. (**Supplementary Figure S2D**) (11). Nevertheless, the snRNA-seq platform offered an additional advantage, as it decouples sample procurement from processing and allows multiplexing of samples collected over time, including biobanked material (24).

Concerning the hepatic myeloid compartment, it remains uncertain whether nuclei can serve as a viable alternative for cellular transcriptomes in the context of advanced liver disease and small sample sizes. This is significant, given that hepatic myeloid cells play a crucial role in both the progression and resolution of tissue inflammation and injury processes (3, 9).

Data analysis of the single nuclei sequenced myeloid cells consistently identify the primary monocyte/macrophage identities found in cirrhotic livers, including Kupffer cells, LAM, and monocytes (3). However, it is important to note that the scRNA-seq platform failed to capture sufficient numbers of Kupffer cells, the most prominent resident macrophage population in the healthy liver. This observation may be elucidated by the reduced presence of Kupffer cells in cirrhotic livers in combination with the limited absolute count of myeloid cells in our study. Additionally, scRNA-seq tends to favor cells that undergo easy dissociation, potentially contributing to the limited representation of myeloid cells in the dataset (3). Generally, snRNA-seq captured a higher percentage of macrophages compared to scRNA-seq, but a lower percentage of monocytes. The data further indicated that the gene signature of the myeloid subclusters was largely preserved in both snRNA-seq and scRNA-seq, with high mean prediction scores and good Pearson correlation coefficients when comparing both techniques. In scRNA-seq, we observed an increase in the expression of ribosomal and mitochondrial RNA, while in snRNA-seq, the expression of long non-coding RNA was notable. However, the most significant difference between both techniques was the heightened dissociation-induced stress signature in scRNA-seq, evident both at the gene level and in pathway analysis. This phenomenon may be attributed to the distinct methodologies employed in snRNA-seq and scRNA-seq. In snRNA-seq, cells undergo rapid freezing and mechanical dissociation, resulting in their swift demise. In contrast, scRNA-seq involves keeping the cells alive during enzymatic dissociation until loading, affording them an opportunity to develop a stress-response. This difference in treatment timelines could contribute to the observed variations in cellular outcomes between the two technique. Our findings strongly indicate that scRNA-seq is less suitable for studying the hepatic myeloid cell landscape in transjugular liver biopsies (TJB) from decompensated cirrhosis patients (3).

In summary, our data strongly suggests that snRNA-seq is superior in recapitulating the hepatic landscape without extensive population bias. The snRNA-seq platform also overcomes challenges related to streamlining clinical specimen collection and downstream experimental procedures, as the procedure can be performed on frozen tissue. Additionally, our results indicate that single-nucleus transcriptome sequencing is the platform of choice for studying myeloid cell populations, as scRNA-seq failed to recover Kupffer cells, and the remaining monocytes/macrophages exhibited increased expression of dissociation-induced stress parameters. Taken together, our data provide essential insights to be considered when undertaking similar sequencing experiments in advanced human cirrhosis.

## Data availability statement

Raw sequence data has been deposited at the European Genome-phenome Archive (EGA), under accession number EGAS50000000073. This study did not generate any new code.

## Ethics statement

The studies involving humans were approved by University Hospitals Leuven ethical committee. The studies were conducted in accordance with the local legislation and institutional requirements. The participants provided their written informed consent to participate in this study. Written informed consent was obtained from the individual(s) for the publication of any potentially identifiable images or data included in this article.

## Author contributions

LM: Conceptualization, Data curation, Formal Analysis, Funding acquisition, Investigation, Methodology, Project administration, Resources, Software, Validation, Visualization, Writing – original draft, Writing – review & editing. JV: Conceptualization, Funding acquisition, Investigation, Methodology, Resources, Supervision, Visualization, Writing – original draft, Writing – review & editing. DB: Conceptualization, Data curation, Formal Analysis, Methodology, Software, Visualization, Writing – original draft. MB: Data curation, Formal Analysis, Methodology, Software, Visualization, Writing – review & editing. BB: Data curation, Methodology, Software, Writing – review & editing. RF-A: Investigation, Visualization, Writing – review & editing. LS: Investigation, Writing – review & editing. MW: Investigation, Project administration, Writing – review & editing. EC: Investigation, Methodology, Writing – review & editing. LB: Investigation, Methodology, Writing – review & editing. GM: Investigation, Methodology, Resources, Supervision, Writing – review & editing. OG: Conceptualization, Investigation, Methodology, Supervision, Visualization, Writing – review & editing. HK: Conceptualization, Formal Analysis, Funding acquisition, Investigation, Methodology, Project administration, Supervision, Visualization, Writing – original draft, Writing – review & editing. DL: Conceptualization, Data curation, Funding acquisition, Investigation, Methodology, Project administration, Resources, Supervision, Writing – original draft, Writing – review & editing. SM: Conceptualization, Formal Analysis, Funding acquisition, Investigation, Methodology, Project administration, Resources, Supervision, Visualization, Writing – original draft, Writing – review & editing.

## Supplementary material

Supplementary Figure 1 Integration of both techniques and patients. **(A)** Annotated UMAP plot of 31,410 single nuclei, split per patient. **(B)** Annotated UMAP plot of 6,152 single cells, split per patient. **(C)** Annotated UMAP plot of 31,410 single nuclei, showing the different clusters. **(D)** Annotated UMAP plot of 6,152 single cells, showing the different clusters.

Supplementary Figure 2 Comparison of gene signatures in major clusters. **(A)** Barplot showing the mean prediction identity score per (sub)cluster of nuclei, predicted using the gene signature of the scRNA-seq data. Score ranging from 0 to 1. Calculated using Seurat-package (FindTransferAnchors, TransferData). **(B)** Barplot showing the mean prediction identity score per (sub)cluster of cells, predicted using the gene signature of the snRNA-seq data. Score ranging from 0 to 1. Calculated using Seurat-package (FindTransferAnchors, TransferData). **(C)** Barplot comparing the stress signature between both techniques in the major celltypes. Significance calculated using a Wilcoxon rank-sum test. **(D)** Dotplot showing the expression of T-cell and B-cell receptor genes in different techniques. The size of the dot shows the percentage of cells expressing the gene and the color the strength of expression.

Supplementary Table 1 Clinical characteristics of patients.

Supplementary Table 2 Number of reads and saturation per sample.
