# Supplementary figures and images for: Comparison of the single-cell and single-nucleus hepatic myeloid landscape within decompensated cirrhosis patients
Source: Front Immunol. 2024 Feb 6;15:1346520. doi: 10.3389/fimmu.2024.1346520 (PMC10878168; doi:10.3389/fimmu.2024.1346520)

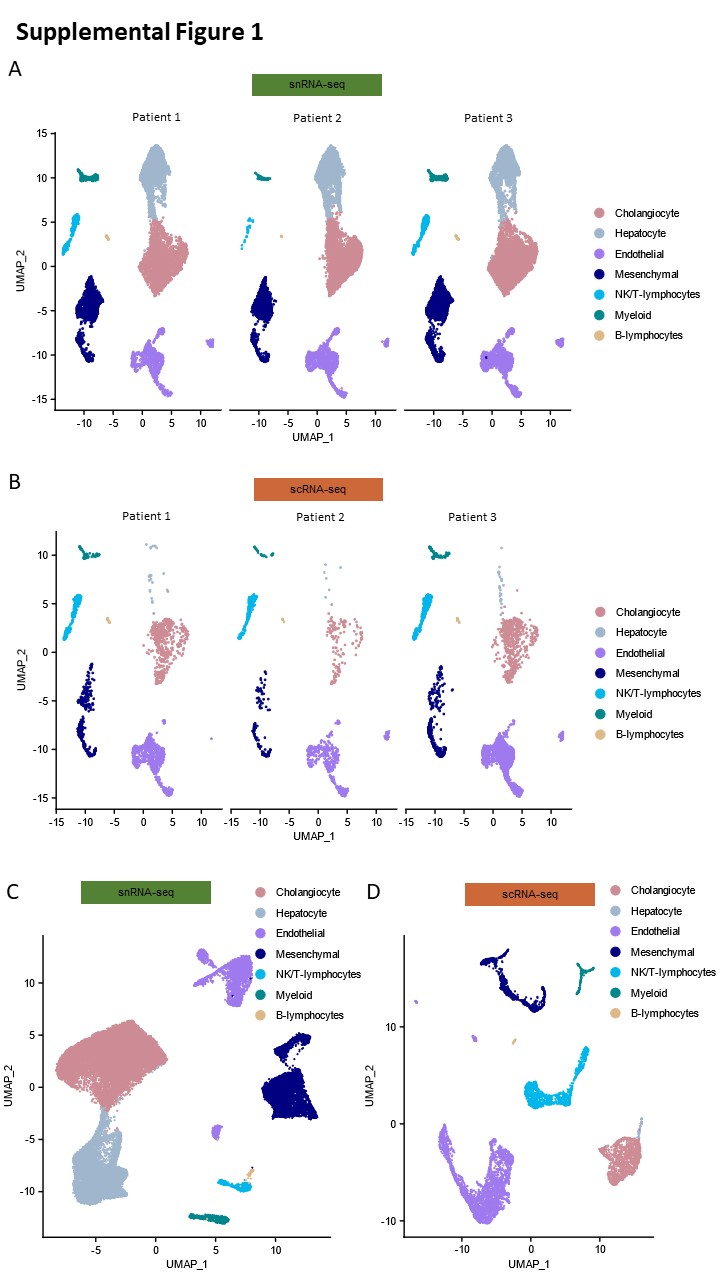

Supplement: Supplementary Figure 1 — Integration of both techniques and patients. (A) Annotated UMAP plot of 31,410 single nuclei, split per patient. (B) Annotated UMAP plot of 6,152 single cells, split per patient. (C) Annotated UMAP plot of 31,410 single nuclei, showing the different clusters. (D) Annotated UMAP plot of 6,152 single cells, showing the different clusters. [file Image_1.jpg]

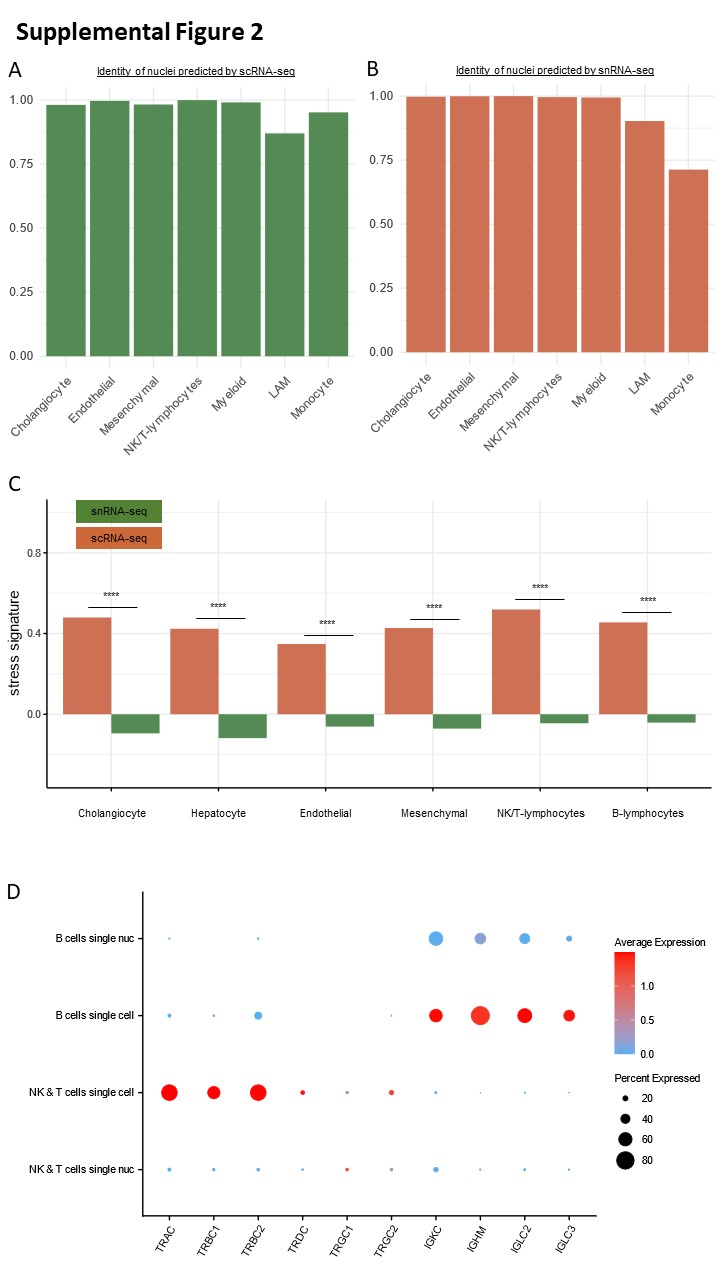

Supplement: Supplementary Figure 2 — Comparison of gene signatures in major clusters. (A) Barplot showing the mean prediction identity score per (sub)cluster of nuclei, predicted using the gene signature of the scRNA-seq data. Score ranging from 0 to 1. Calculated using Seurat-package (FindTransferAnchors, TransferData). (B) Barplot showing the mean prediction identity score per (sub)cluster of cells, predicted using the gene signature of the snRNA-seq data. Score ranging from 0 to 1. Calculated using Seurat-package (FindTransferAnchors, TransferData). (C) Barplot comparing the stress signature between both techniques in the major celltypes. Significance calculated using a Wilcoxon rank-sum test. (D) Dotplot showing the expression of T-cell and B-cell receptor genes in different techniques. The size of the dot shows the percentage of cells expressing the gene and the color the strength of expression. [file Image_2.jpg]
