# Supplementary material for: Comparison of the single-cell and single-nucleus hepatic myeloid landscape within decompensated cirrhosis patients
Source: Front Immunol. 2024 Feb 6;15:1346520. doi: 10.3389/fimmu.2024.1346520 (PMC10878168; doi:10.3389/fimmu.2024.1346520)
Supplement: Supplementary Table 1 — Clinical characteristics of patients. [file Table_1.docx]

**Supplemental table 1: Clinical characteristics of patients**

| **Patient** | **Age** | **Gender** | **BMI**  **(kg/m^2^)** | **Pathology** | **Ultrasound** | **MELD-**  **score** | **ACLF-**  **grade** | **Liver disease** |
| --- | --- | --- | --- | --- | --- | --- | --- | --- |
| 1 | 51 | Male | 25.1 | Micronodular cirrhosis | Cirrhotic liver, ascites | 17 | 0 | ALD |
| 2 | 35 | Female | 35.2 | Micronodular cirrhosis | Cirrhotic liver, no ascites | 27 | 2 | ALD/MAFLD |
| 3 | 51 | Male | 30.4 | Micronodular cirrhosis | Cirrhotic liver, ascites | 18 | 0 | ALD/MAFLD |

*ACLF-grade: acute-on-chronic liver failure (European foundation for the study of chronic liver failure definition); ALD : alcohol-related liver disease; BMI : body mass index; MAFLD : metabolic-associated fatty liver disease; MELD : model for end stage liver disease*
