# Supplementary material for: Comparison of the single-cell and single-nucleus hepatic myeloid landscape within decompensated cirrhosis patients
Source: Front Immunol. 2024 Feb 6;15:1346520. doi: 10.3389/fimmu.2024.1346520 (PMC10878168; doi:10.3389/fimmu.2024.1346520)
Supplement: Supplementary Table 2 — Number of reads and saturation per sample. [file Table_2.docx]

**Supplemental table 2: Number of reads and saturation per sample**

| **Patient** | **Technique** | **Number of reads** | **Saturation** | **Viability*** | **Post-rho**** | **Number of nuclei/cells***** | **Number of nuclei/cells****** |
| --- | --- | --- | --- | --- | --- | --- | --- |
| 1 | Single-nucleus RNA-sequencing | 390,684,435 | 42.7% | 2% | 0.04 | 9,871 | 7,119 |
| 1 | Single-cell RNA-sequencing | 217,571,095 | 52.4% | 81% | 0.05 | 6,322 | 1,919 |
| 2 | Single-nucleus RNA-sequencing | 355,222,721 | 47.7% | 1% | 0.10 | 14,997 | 9,758 |
| 2 | Single-cell RNA-sequencing | 115,068,133 | 59.2% | 90% | 0.03 | 2,088 | 933 |
| 3 | Single-nucleus RNA-sequencing | 461,285,033 | 40.4% | 1% | 0.08 | 19,839 | 14,178 |
| 3 | Single-cell RNA-sequencing | 287,433,249 | 57.0% | 82% | 0.06 | 7,698 | 3,308 |

** As measured by LUNA-FL before generating the libraries. Target > 70% for scRNA-seq, <5% for snRNA-seq
** As a measure for the estimated contamination fraction, calculated using the soupX package in R. Mean Post-rho was not significantly different between the two techniques (p=0.37 on paired t-test).
*** After correction for empty droplets (via Cellranger)
****After quality control and doublet removal*
