# Supplementary material for: Comparison of the single-cell and single-nucleus hepatic myeloid landscape within decompensated cirrhosis patients
Source: Front Immunol. 2024 Feb 6;15:1346520. doi: 10.3389/fimmu.2024.1346520 (PMC10878168; doi:10.3389/fimmu.2024.1346520)
Supplement: Supplementary file 5 [file DataSheet_1.docx]

**Supplemental Methods**

**Buffers**

ST-buffer was made with nuclease free water with 96mM NaCl, 10mM Tris-HCl pH 7.5, 1mM CaCl_2_, 21mM MgCl_2_ and 0.1 units/µl of Rnasin-Plus (ThermoFisher Scientific). TST-buffer was made with nuclease free water with 96mM NaCl, 10mM Tris-HCl pH 7.5, 1mM CaCl_2_, 21mM MgCl_2_, 0.03% of Tween-20, 0.01% of Bovine Serum Albumin and 0.1 units/µl of Rnasin-Plus (ThermoFisher Scientific).
